# Computer-assisted image-based risk analysis and planning in lung surgery - a review

**DOI:** 10.3389/fsurg.2022.920457

**Published:** 2022-09-22

**Authors:** Stefan Krass, Bianca Lassen-Schmidt, Andrea Schenk

**Affiliations:** ^1^ Fraunhofer Institute for Digital Medicine MEVIS, Bremen, Germany; ^2^ Department of Diagnostic and Interventional Radiology, Hannover Medical School, Hannover, Germany

**Keywords:** image processing, lung surgery, risk analysis, surgical planning, lung cancer, quantification

## Abstract

In this paper, we give an overview on current trends in computer-assisted image-based methods for risk analysis and planning in lung surgery and present our own developments with a focus on computed tomography (CT) based algorithms and applications. The methods combine heuristic, knowledge based image processing algorithms for segmentation, quantification and visualization based on CT images of the lung. Impact for lung surgery is discussed regarding risk assessment, quantitative assessment of resection strategies, and surgical guiding. In perspective, we discuss the role of deep-learning based AI methods for further improvements.

## Introduction

Computational image-based surgical planning and risk analysis has been the subject of research during the last decades. Different approaches were developed in particular for brain, lung and liver surgery. For lung surgery, different imaging modalities, e.g. fluorescence bronchoscopic technique (1), as well as a variety of other devices (2) exist. In this paper, we concentrate on CT-based modelling of the patient individual lung morphology combined with quantification and visualization methods to support thoracic surgeons prior and during surgical interventions.

Chen-Yoshikawa et al. (3) give an overview about current trends in thoracic surgery and present, among other topics, the current role of CT-based image analysis and modeling for planning and risk analysis in thoracic surgery. They still see a limitation in the complexity in manipulating the discussed software systems. Matsumoto et al. (4) compare different software programs for the visualization of anatomical lung structures based on volume and surface rendering. They concluded that the generated three-dimensional (3D) images facilitate a better understanding of anatomic structures. They also see a lack in accuracy in subsegmental blood vessels compared to intraoperative findings. Ikeda et al. (5) outline the importance of knowledge about the anatomy prior and during surgery, in particular for video-assisted throracoscopic surgery (VATS) and evaluate a particular software system (Synapse Vincent, Fuji Film Co., Ltd., Tokyo, Japan). They discuss in particular the role of 3D image analysis for the knowledge of the patient individual blood vessel structure and see impact on safety and education. Nia et al. (6) use this system for preoperative planning and guiding of video-assisted surgery and conclude that preoperative planning with interactive 3D CT reconstruction is useful to prepare a surgeon with knowledge on specific anatomic variations. They also demonstrate the feasibility of intraoperative 3D guidance in VATS. Also (7) retrospectively evaluated the impact of 3D modeling of the pulmonary vessels on lung resection. They focused on robotic interventions and emphasize the improvement of confidence of the surgeon in recognizing anatomical structures and the appreciation of pulmonary artery variations.

Chen et al. (8) describe the role of computed-tomography based reconstruction of the lung morphology as a basis for further applications of virtual and augmented reality. They demonstrate the ability to segment vessels and bronchi as well as the tumor (incl. safety margin) based on CT data with the commercial workstation Ziostation (Amin, Inc., Tokyo, Japan). Based on the same techniques, Iwano et al. (9) demonstrated the impact on segmentectomy of the lung.

In Tokuno et al. (10), a new approach for virtual anatomic reconstruction was presented, that considers the deformation of the lung during the intervention. Lesage et al. (11) evaluated the use of modeling of pneumothorax to predict the tumor localization during minimal invasive surgery based on a preoperative CT scan.

Sortini et al (12) give an overview on intra-thoracoscopic localization techniques to improve the detectability of smaller pulmonary nodules during intervention. The review describes a variety of different invasive peroperative techniques like radioguiding vital dyes, and hook wires. They conclude that there is no superiority of one specific technique but see advantages of ultrasound guidance compared to invasive techniques due to reduced complications.

Strength and limitations: The work presented in this paper emphasizes the importance of explicit segmentation of anatomical structures of the lung in computed tomographic images followed by quantification algorithms and adequate visualization techniques to support the thoracic surgeon. In contrast to methods that purely rely on volume rendering techniques, this explicit segmentation allows for quantification-based risk analysis, as will be shown later. First methods for the determination of bronchopulmonary segments based on CT were described 2000 by Krass et al. (13) and subsequently validated by Böhm et al. (14). Using this approach, methods for functional analysis of lungs, lung lobes, and bronchopulmonary segments where developed by Kuhnigk et al. (15).

## Methods

### Segmentation

Based on lung CT data, we have developed methods for the segmentation of even complex tumors in the lung, methods for the segmentation and labeling of the bronchial tree, for the pulmonary vessel system, and, what is expected to be important for anatomic resection strategies, the determination of bronchopulmonary lobes and segments. Related work will be found within the respective cited literature.

#### Tumor segmentation

**Figure 1:**
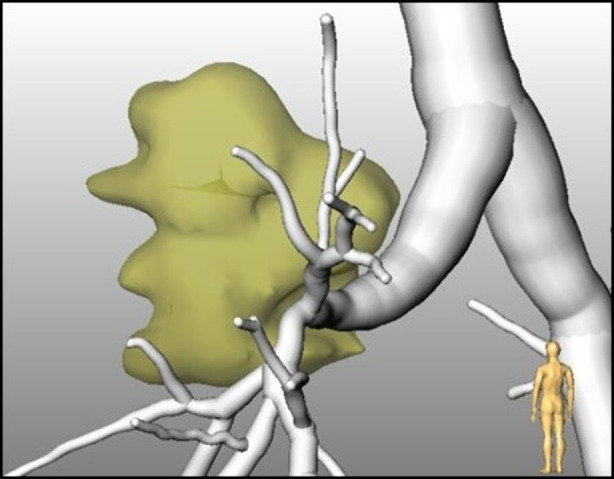
CT-based segmentation of a tumor in relation to the bronchial tree.

Tumors are segmented with a semi-automated algorithm that calculates, after an interactive selection of the tumor position, the borders of the tumor based on morphological assumptions. Adjacent vessels are automatically excluded from the segmentation result. The tumor segmentation is robust even in complex anatomical regions, but still needs an interactive selected starting point within the tumor. Figure 1 shows an example of a segmented tumor. The methods – as well as related work - are described in more detail in Kuhnigk et al. (16).

#### Bronchial tree

**Figure 2:**
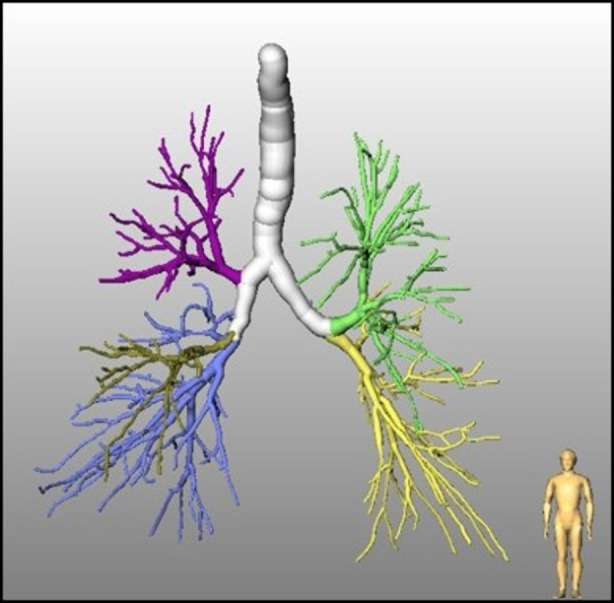
Bronchial tree with automatically identified lobe bronchi.

An adaptive region growing method initially proposed by Selle et al. (17) for liver vessels is used for the segmentation of the bronchial tree. It automatically adapts the segmentation threshold with the increase of the segmented airways. For details, we refer to Zidowitz et al. (18) and Schmidt et al. (19). In Figure 2 the result of a CT-based airway segmentation is shown. The algorithm also enables fully automated labeling of the bronchi according to their affiliation with the individual bronchopulmonary lobes.

#### Blood vessels

**Figure 3:**
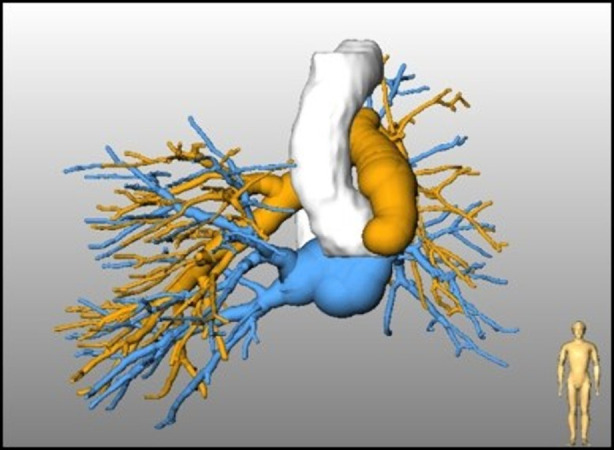
Pulmonary arteries and veins in relation to bronchial tree.

Due to the high contrast, segmentation of blood vessels can be done using a conventional 3D region growing algorithm. This fully automated approach includes an automatic detection of seed points in the hilum region (15). The segmentation of blood vessels within the lungs is not able to differentiate between pulmonary arteries and veins. The method was part of the “VESSEL 12” challenge (VESsel SEgmentation in the Lung 2012) (20) that was closed in November 2019 and is still on rank two among 31 submissions. A separation of pulmonary veins and arteries was achieved by an additional interaction by marking specific points in veins and arteries. A result of the overall procedure is shown in Figure 3.

#### Lobes and segments

**Figure 4:**
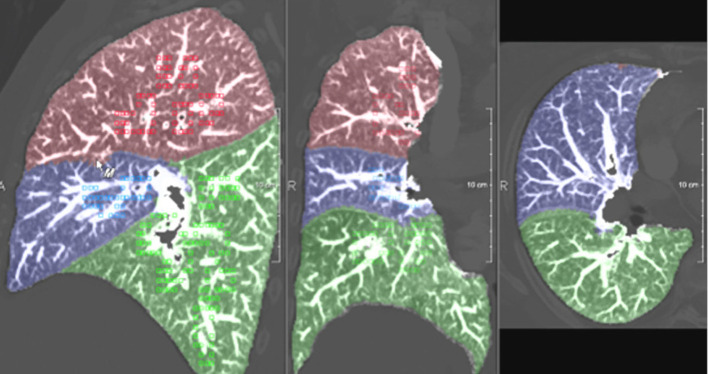
Automatically identified lung lobes.

Lobar boundaries are detected by a hybrid approach that generates a cost image based on the absence or rareness of larger blood vessels adjacent to the boundary, and an original image, where segmented blood vessels are removed (15). This approach was expanded by Lassen-Schmidt et al. (21) who also included an explicit segmentation of the pulmonary fissures (see Figure 4) (22). This method participated in an international challenge (LOLA11, LObe and Lung Analysis 2011) (23), where it was ranked first at the time of the challenge.

**Figure 5:**
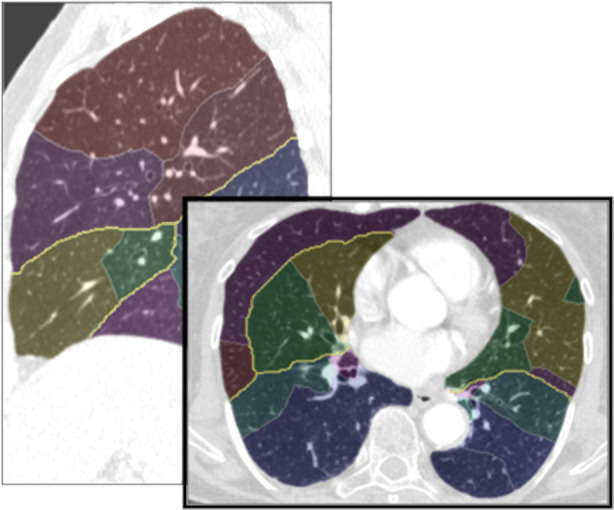
Identified bronchopulmonary segments.

The original approach for segmentation of pulmonary segments (13) was based on the segmented bronchial tree and therefore limited in accuracy. Validation studies yield an accuracy of volumetric overlap between segmentation and ground truth of approxemately 80%. A sufficient determination of the borders of pulmonary segments is not possible with this method. Therefore, Stöcker et al. proposed a segment segmentation, that is based on pulmonary arteries, and achieved an accuracy of 2–3 mm compared to groundtruth for the localization of segment boundaries computed by the pulmonary artery-based method (23). A result of this segment approximation based on CT data is shown in Figure 5.

### Quantification

Using segmentations, the quantification of a variety of measures which may assist planning or clinical decision support become possible: tumor volume, tumor distances – to lobe or segmental borders, but also to the different bifurcations of the bronchial tree. Furthermore, based on the lobe and segment segmentation, it is not only possible to determine the volume of each individual anatomic unit of the lung, but it also enables the computation of CT parameters, that characterize the morphology and function of the parenchyma – like mean lung density or emphysema index. These quantification methods, which rely strongly on explicit segmentation of morphologic structures of the lung, are a prerequisite for risk analysis prior to lung surgery.

#### Tumor morphology

Tumor volume quantification is possible on the basis of the segmentation. The algorithm takes also into account partial volume effects at the boundary of the tumor. This is of particular importance with smaller tumors, where border voxels account for more than 50% of the total volume. More details about the volumetric method can be found in (16).

**Figure 6:**
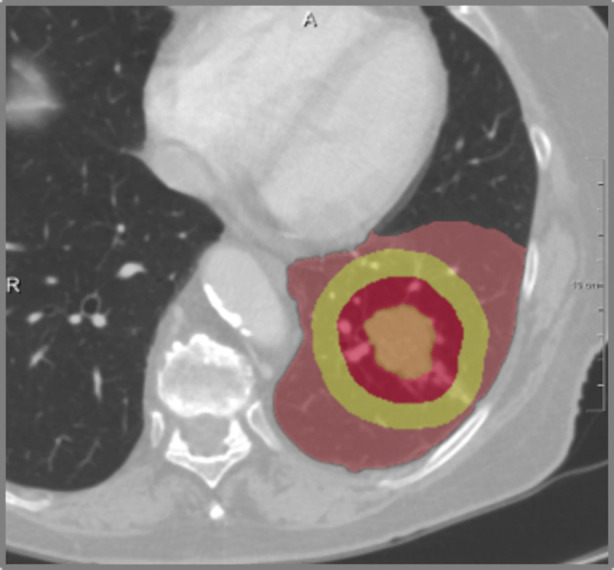
Safety margin between tumor and segment border.

**Figure 7:**
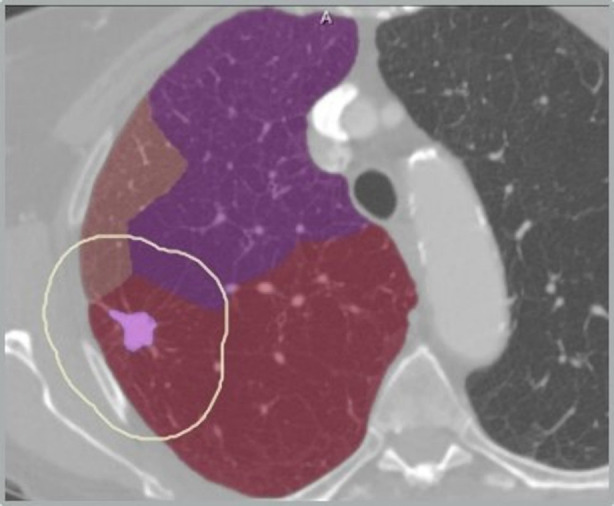
Distance between tumor and segment border (2 mm).

Beside tumor volume, quantification of tumor distances to specific anatomic landmarks is possible based on the segmented lung morphology. In particular distances to carina and to lobe and segment borders are of prognostic value for tumor resection and allow for infiltration risk assessment related to different resection strategies - like lobe resection vs. (bi-)segmentectomy. The methods and application for distance-based risk analysis are described in detail in (24, 25). Figure 6 shows an example of a tumor safety margin in relation to the boundary of a brochopulmary segment. Quantification of distance between tumor and segment border is demonstrated in Figure 7. Use of pulmonary arteries instead the bronchial tree for the identification of bronchopulmonary segment boarders is essential. Even with this bronchopulmonary segment boarders are identified with an accuracy of about 2 mm. Part of future work is the incorporation of pulmonary veins, that often are located between segment boarders.

#### Lobe and segment-based quantification

**Figure 8:**
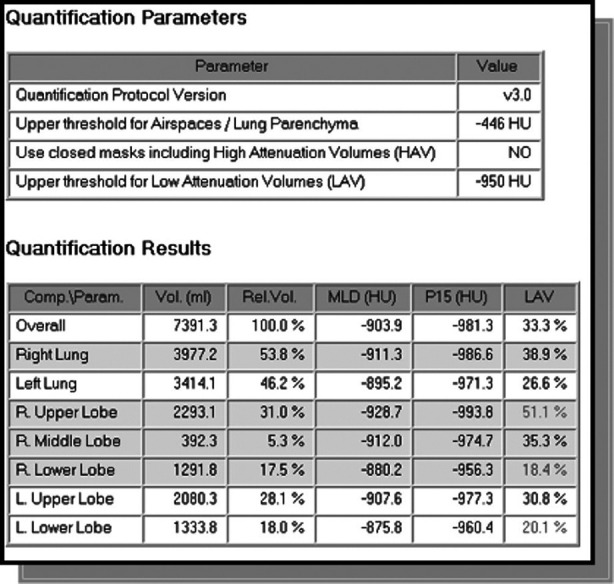
Lobe based functional CT parameters.

Based on the segmentation of pulmonary lobes and segments, the calculation of quantitative CT parameters, like volume, volume percentage, mean lung density, percentiles, and low – as well as – high attenuation values become possible for each anatomical lung unit. This allows for a differentiated analysis of lung structure. Figure 8 shows an example of lobe-based quantification of lung CT parameters.

### Visualization

**Figure 9:**
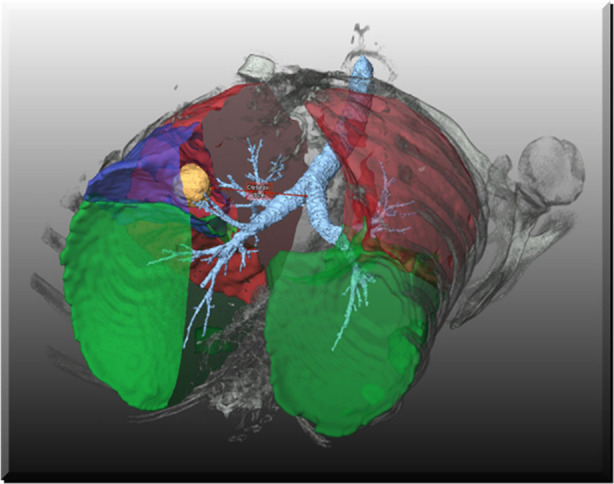
Visualization of segmented bronchi, lobe borders and tumor.

**Figure 10:**
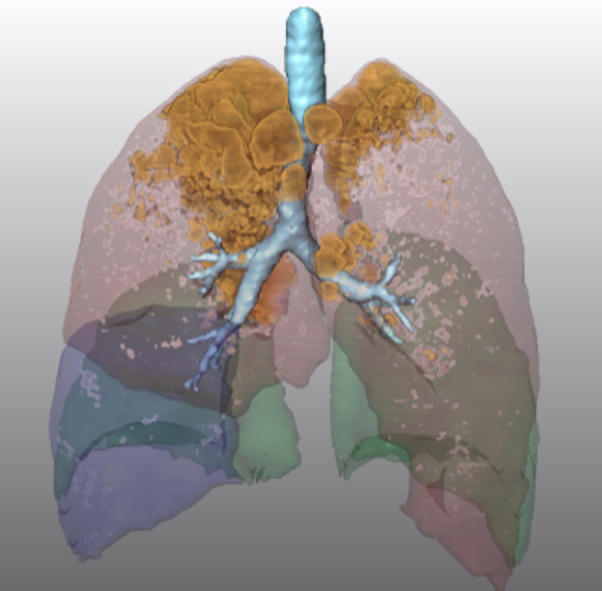
Visualization of segmented bronchopulmonary borders and emphysematous regions.

The segmentation of lung structures in CT images enables a highly selective visualization of the lung morphology, e. g. bronchopulmonary lobes, bronchial tree and tumor or extended emphysema in specific lobes. This supports the surgeons in the planning phase prior to, as well as during, the intervention as a guiding tool (26). Figure 9 demonstrates the selective visualization of lung lobe borders, tumor and bronchi. In Figure 10, the relation between emphysematous regions and lung lobes is visualized.

**Figure 11:**
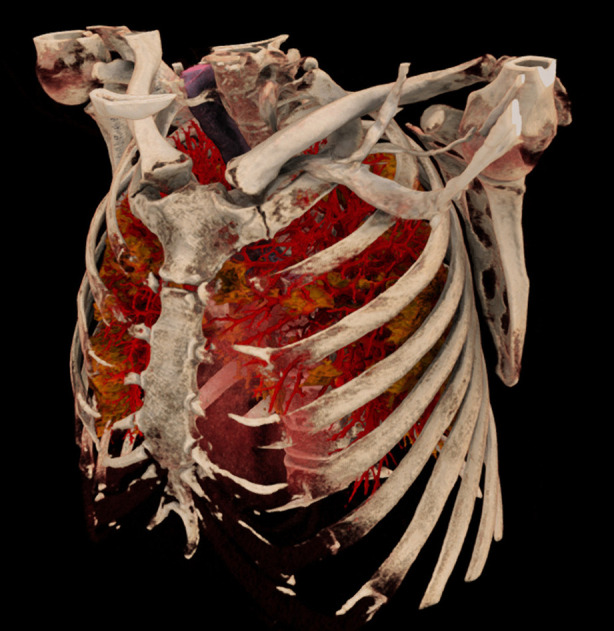
AVIS rendering technique.

The visualization of segmented structures as opaque or semi-transparent surface renderings is also directly suited for display in augmented and virtual reality devices. It can be combined with basic direct volume renderings of the CT data, as shown, e.g., for the bones in Figure 9. Using recent fast approaches of direct volume rendering like adaptive volumetric illumination sampling (AVIS) (27), a highly realistic visualization of the lung and it structures becomes feasible (Figure 11), even for augmented reality devices that may support the surgeon in the operation room.

## Expected impact for lung surgery

Impact of computer-assistance for lung surgery is expected within the three categories: general operability, resection strategy, and surgical planning and guidance. These three categories will be discussed in the following paragraphs.

### Operability

There are two prominent limitations for the operability of a patient. One is the expected remaining lung function after resections, the other limitation is the probability of tumor infiltration in larger adjacent lung structures like blood vessels or pericardium.

An estimation of postoperative lung function is often performed using 2D projection scintigraphy. With this additional invasive method, 3D information is not available. In contrast, CT-based quantification of remaining volumes of the lung parenchyma combined with lobe-based quantification of CT parameters like mean lung density, emphysema index, emphysema classification and fibrosis index may allow for a better prognosis of post-operative lung function. The determination of CT parameters is described in (28), the approximation of post-operative lung function in (15).

The direct diagnosis of tumor infiltration into neighboring structures is challenging and often not possible purely based on CT data. Nevertheless, the distance between tumor border and adjacent structures could indicate the infiltration probability. Limmer et al. (29, 30) show surgical examples, where CT-based morphologic features allow for the estimation of infiltration probability.

### Resection strategy

**Figure 12:**
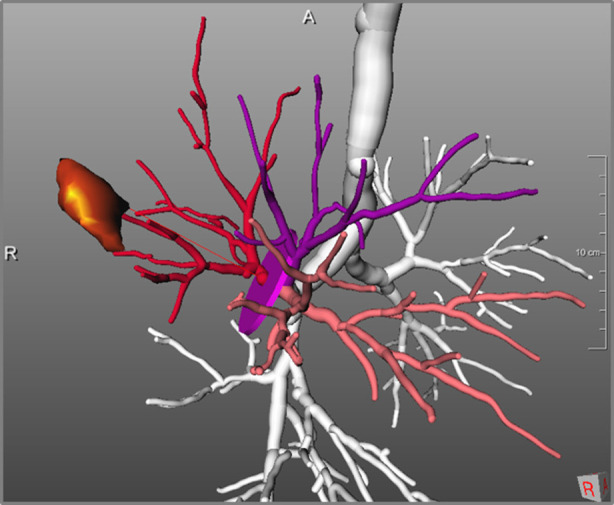
Determination of distance between tumor and resection cutting point of bronchial tree: 51 mm.

The second expected impact of computer-assistance for thoracic surgery is the support for choosing an adequate resection strategy for an individual patient. A combination of quantitative morphological parameters - like size and form of tumor, distance to carina and lobe bronchus, or spatial relationships to lobes and segments, combined with functional quantitative CT parameters, can impact decision making regarding resection strategies like lobectomy, segmentectomy or bi-segmentectomy. This refers to different extents of resections, but also to different surgical techniques (31, 32). Key parameters for the planning of segment resections are tumor size, distance to segment border, distance to the arterial and bronchial cutting point, and safety margins (for example visualizations see Figures 6, 7, 12).

### Surgical planning and guidance

The third expected impact on thoracic surgery is based on visualization techniques and should help the surgeon *via* image-based guidance. Examples are the visualization of pulmonary metastasis which can guide the surgeon during metastatic surgery or the visualization of a central tumor in relation to bronchi and pulmonary vessel systems in particular for video-assisted throracoscopic surgery (26, 29).

## Commonalities with liver surgery planning

Some approaches for surgical planning and risk analysis for lung share commonalities with liver surgical planning. Therefore, we also give a short review over related methods to support liver surgery in oncological cases as well as in living donor liver transplantation.

As for lung surgery, the first step is the segmentation of basic anatomic, pathologic and potential risk structures from contrast-enhanced CT or magnetic resonance imaging (MRI) data. For liver, these structures are the organ itself, the hepatic vessel systems of liver artery, portal vein, and hepatic vein as well as lesions in case of planning for oncologic surgery. Bile ducts can be segmented and integrated if they were imaged using a particular contrast agent (Iotroxic Acid; limited availability) or additional image sequences (e.g. magnetic resonance cholangiopancreatography, MRCP). Segmentation of the liver and hepatic lesions has recently switched from using established image analysis methods to deep learning approaches [e.g. (33, 34)]. A fact which was also confirmed by the international LiTS (Liver Tumor Segmentation) challenge in 2017 (35, 36) where all algorithms that placed in the top 10 used deep neural networks. Vessel segmentations are still a matter of traditional image processing approaches using a region growing step (e.g. 17, 37), but first deep learning networks have also been presented for this task (38, 39).

Risk analysis and planning for liver surgery is typically based on perfusion and drainage territories and their combination with virtual resections. Perfusion territories can be a schematic division of the liver according to the Couinaud segments (40) and can be transferred using landmarks or applying a DL network, but can alternatively and more precisely be computed from the individual branches of the portal vein system (e.g. 17). Drainage territories estimated from the hepatic vein branches can be of great importance in particular for major resections such as extended hemi-hepatectomies and in living donor liver transplantations. Studies have shown that computer-assisted surgical planning and risk analysis can change surgery in about one third of complex cases when compared to conventional surgical planning (41, 42). In these challenging surgical cases, not only liver volume but also hepatic function plays an important role and combinations thereof can further improve the preoperative risk analysis and outcome prediction (43).

## Current work and outlook

The acceptance of the presented methods in clinical routine relies on the grade of automation and robustness with regard to different image types for the processing of the CT images. The effort to achieve the desired image processing results should be feasible regarding time and interaction. For currently used heuristic, knowledge-based methods, there is a highly grade of automation for segmenting bronchi, lungs, lobes and tumors. However, automatic segmentation of lungs and lobes in cases where the patients suffer from lung infiltration is limited. Also, the separate segmentation of pulmonary arteries and veins is a tedious task. The change of paradigms in medical image computing that took place during recent years in the transition from heuristic, knowledge-based algorithms to deep learning methods gives a chance to enhance automation and adaption to different image data types, as well as apply these approaches for more complex procedures which were beyond the previous methods.. With this the clinical uptake of new methods could be far quicker and easier than before.

In (44), a broad overview over the developments in computer analysis in chest imaging during the last decades is given. The author describes the transition to deep learning-based algorithms. Examples are presented for airway and fissure segmentation, nodule detections and classification. Deep convolutional networks are seen as the technique of choice for image analysis. Proper implementation of software for building and training such networks is emphasized. A review on deep learning based structural and functional analysis across a variety of lung imaging modalities is given in (45). The authors give an overview of the deep learning research literature with regard to lung image analysis applications. Lassen-Schmidt et al. (46) present the automatic segmentation of the pulmonary lobes with a 3D u-net and optimized loss function.

There are several advantages of deep learning-based methods over knowledge-based algorithms. Deep learning networks learn from data characteristics that maybe difficult to recognize by humans. Adaptions of models to different scanners, resolutions and contrast agents are easier to perform, because new data can be included into the training set or added by transfer learning. If annotated data exist, also the segmentation of new structures is applicable and faster compared to classical heuristic approaches.

Drawbacks are the need of a powerful hardware infrastructure for the training of the algorithms. Of significant importance is the need of a large number of datasets including respective annotation which, at present, limits the development of deep learning-based algorithms. A third drawback may be the risk of overfitting if the training datasets do not cover the variety of anatomical variations and differences in imaging techniques and in the routine data to which the algorithm is later applied.

For image-based planning and risk analysis in lung surgery, we anticipate that in the short term deep learning-based methods applied to an automated separation of pulmonary veins and arteries and a more robust lung and lobe segmentation in high pathological cases will be impactful.

## References

[1] Moghissi K Dixon K. Image-guided surgery and therapy for lung cancer: a critical review. Future Oncol. (2017) 13(26):2383–94. 10.2217/fon-2017-0265 29129114

[2] Zhao Z Jordan S Tse ZTH. Devices for image-guided lung interventions: state-of-the-art review. Proc Inst Mech Eng H. (2019) 233(4):444–63. 10.1177/0954411919832042 30843465

[3] Chen-Yoshikawa TF Fukui T Nakamura S Ito T Kadomatsu Y Tsubouchi H Current trends in thoracic surgery. Nagoya J Med Sci. (2020) 82(2):161–74. 10.18999/nagjms.82.2.161 32581397 PMC7276403

[4] Matsumoto T Kanzaki M Amiki M Shimizu T Maeda H Sakamoto K Comparison of three software programs for three-dimensional graphic imaging as contrasted with operative findings. Eur J Cardiothorac Surg. (2012) 41:1098–103. 10.1093/ejcts/ezr152 22219443

[5] Ikeda N Yoshimura A Hagiwara M Akata S Saji H. Three dimensional computed tomography lung modeling is useful in simulation and navigation of lung cancer surgery. Ann Thorac Cardiovasc Surg. (2013) 19(1):1–5. 10.5761/atcs.ra.12.02174 23364234

[6] Nia P S Olsthoorn JR Heuts S Maessen JG. Interactive 3D reconstruction of pulmonary anatomy for preoperative planning. Virtual Simul Intraoper Guiding Video-Assist Thoracoscopic Lung Surg. (2019) 14(1):17–26. 10.1177/1556984519826321 30848710

[7] Bhakhri K Hyde ER Sze MM Berger LU Ourselin S Routledge T Surgeon knowledge of the pulmonary arterial system and surgical plan confidence is improved by interactive virtual 3D-CT models of lung cancer patient anatomies. Front Surg. (2021) 8:652428. 10.3389/fsurg.2021.652428 33855044 PMC8040802

[8] Chen Z Zhang Y Yan Z Dong J Cai W Ma Y Artificial intelligence assisted display in thoracic surgery: development and possibilities. J Thorac Dis. (2021) 13(12):6994–7005. 10.21037/jtd-21-1240 35070382 PMC8743398

[9] Iwano S Yokoi K Taniguchi T Kawaguchi K Fukui T Naganawa S. Planning of segmentectomy using three-dimensional computed tomography angiography with a virtual safety margin: technique and initial experience. Lung Cancer. (2013) 81(3):410–5. 10.1016/j.lungcan.2013.06.001 23838090

[10] Tokuno J Chen-Yoshikawa TF Nakao M Matsuda T Date H. Resection process map: a novel dynamic simulation system for pulmonary resection. J Thorac Cardiovasc Surg. (2020) 159(3):1130–8. 10.1016/j.jtcvs.2019.07.136 31606178

[11] Lesage AC Rajaram R Tam AL Rigaud B Brock KK Rice CD Preliminary evaluation of biomechanical modeling of lung deflation during minimally invasive surgery using pneumothorax computed tomography scans. Phys Med Biol. (2020) 65(22):225010. 10.1088/1361-6560/abb6ba 32906090

[12] Sortini D Feo C Maravegias K Carcoforo P Pozza E Liboni A Intrathoracoscopic localization techniques. Review of literature. Surg Endosc. (2006) 20(9):1341–7. 10.1007/s00464-005-0407-z 16703435

[13] Krass S Selle D Boehm D Jend HH Kriete A Rau W A method for the determination of bronchopulmonary segments based on HRCT data. In: Lemke HU Vannier MW Inamura K Farman AG Doi K, editors. Computer assisted radiology and surgery. New York: Elsevier Science (2000). p. 584–9.

[14] Boehm D Krass S Kriete A Rau WS Selle D Jend H-H “Segmentbestimmung im computertomogramm der lunge”: in-vitro validierung. In: Horsch A Lehmann TM, editors. Bildverarbeitung für die medizin. Berlin: Springer (2000). p. 168–72.

[15] Kuhnigk JM Dicken V Zidowitz S Bornemann L Kuemmerlen B Krass S Informatics in radiology (infoRAD): new tools for computer assistance in thoracic CT. Part 1. Functional analysis of lungs, lung lobes, and bronchopulmonary segments. Radiographics. (2005) 25(2):525–36. 10.1148/rg.252045070 15798068

[16] Kuhnigk JM Dicken V Bornemann L Bakai A Wormanns D Krass S Morphological segmentation and partial volume analysis for volumetry of solid pulmonary lesions in thoracic CT scans. IEEE Trans Med Imaging. (2006) 25(4):417–34. 10.1109/TMI.2006.871547 16608058

[17] Selle D Preim B Schenk A Peitgen HO. Analysis of vasculature for liver surgical planning. IEEE Trans Med Imaging. (2002) 21:1344–57. 10.1109/TMI.2002.801166 12575871

[18] Zidowitz S Schmidt A Kriete A Krass S Peitgen H-O. Steps towards a patient individual geometric model of the bronchial tree used for functional simulations. In: Amini AA Manduca A, editors. Proceedings of SPIE, vol. 5369. Bellingham: SPIE (2004). p. 125–31.

[19] Schmidt M Kuhnigk JM Krass S Owsijewitsch M de Hoop B Peitgen H-O. Reproducibility of airway wall thickness measurements. In: Karssemeijer N Summers RM, editors. Proceedings of SPIE, vol 7624. Bellingham: SPIE (2010). p. 76241O. doi: 10.1117/12.844453.

[20] VESsel SEgmentation in the Lung. https://vessel12.grand-challenge.org/ (Accessed April 7, 2022) (2012).

[21] Lassen B van Rikxoort EM Schmidt M Kerkstra S van Ginneken B Kuhnigk JM. Automatic segmentation of the pulmonary lobes from chest CT scans based on fissures, vessels, and bronchi. IEEE Trans Med Imaging. (2013) 32(2):210–22. 10.1109/TMI.2012.2219881 23014712

[22] Lassen-Schmidt BC Kuhnigk JM Konrad O van Ginneken B van Rikxoort EM. Fast interactive segmentation of the pulmonary lobes from thoracic computed tomography data. Phys Med Biol. (2017) 62(16):6649–65. 10.1088/1361-6560/aa7674 28570264

[23] LObe and Lung Analysis. https://lola11.grand-challenge.org/ (Accessed April 7, 2022) (2011).

[24] Stoecker C Welter S Moltz JH Lassen B Kuhnigk JM Krass S Determination of lung segments in computed tomography images using the Euclidean distance to the pulmonary artery. Med Phys. (2013) 40(9):091912. 10.1118/1.4818017 24007163

[25] Stoecker C Welter S Klemm W Beckers F Witte B Krass S. Computer assistance in lung surgery for segment resections and minimally invasive surgery. In: Hahn HK Kikinis R Klein J Nabavi A Weber S, editors. CURAC 2015 Tagungsband. Bremen: Fraunhofer MEVIS (2015). p. 327–32.

[26] Dicken V Kuhnigk JM Bornemann L Zidowitz S Krass S Peitgen H-O. Novel CT data analysis and visualization techniques for risk assessment and planning of thoracic surgery in oncology patients. In: Lemke HU Inamura K Doi K Vannier MW Farman AG, editors. Computer assisted radiology and surgery. Amsterdam: Elsevier Science (2005). p. 783–7.

[27] Kraft V Link F Schenk A Schumann C. Adaptive illumination sampling for direct volume rendering. In: Magnenat-Thalmann N Stephanidis C Wu E Thalmann D Sheng B Kim J Papagiannakis G Gavrilova M, editors. CGI 2020, LNCS 12221. Cham: Springer Nature Switzerland AG (2020). p. 107–18.

[28] Boehm D Krass S Selle D Jend H-H Peitgen H-O. Segmentabhängige bestimmung von quantitativen funktionsparametern aus dem CT der lunge. In: Handels H Horsch A Lehmann T Meinzer HP, editors. Bildverarbeitung für die medizin. Berlin: Springer (2001). p. 295–9.

[29] Limmer S Dicken V Kujath P Krass S Stöcker C Wendt N Three-dimensional reconstruction of central lung tumors based on CT data. Chirurg. (2010) 81(9):833–40. 10.1007/s00104-009-1828-3 19940969

[30] Limmer S Stöcker C Dicken V Krass S Wolken H Kujath P. Computer-Assisted visualization of central lung tumours based on 3-dimensional reconstruction. In: Subburaj K, editors. CT Scanning – techniques and applications. London: InTech (2011). p. 205–28.

[31] Welter S Stöcker C Dicken V Kühl H Krass S Stamatis G. Lung segment geometry study: simulation of largest possible tumours that fit into bronchopulmonary segments. Thorac Cardiovasc Surg. (2012) 60(2):93–100. 10.1055/s-0030-1271009 21695673

[32] Stoecker C Bornemann L Dicken V Krass S Kuhnigk JM Zidowitz S CT-based patient individual anatomical modeling of the lung and its impact on thoracic surgery. In: Dössel O Schlegel WC, editors. IFMBE Proceedings, vol 25/IV. Berlin: Springer (2009). p. 1592–5.

[33] Chlebus G Schenk A Moltz JH van Ginneken B Hahn HK Meine H. Automatic liver tumor segmentation in CT with fully convolutional neural networks and object-based postprocessing. Sci Rep. (2018) 8:15497. 10.1038/s41598-018-33860-7 30341319 PMC6195599

[34] Chlebus G Meine H Thoduka S Abolmaali N van Ginneken B Hahn HK Reducing inter-observer variability and interaction time of MR liver volumetry by combining automatic CNN-based liver segmentation and manual corrections. PLoS ONE. (2019) 14(5):e0217228. 10.1371/journal.pone.0217228 31107915 PMC6527212

[35] LiTS - Liver Tumor Segmentation Challenge. https://competitions.codalab.org/competitions/17094/ (Accessed April 14, 2022) (2017).

[36] Bilic P Christ PF Vorontsov E Chlebus G Chen H Dou Q The Liver Tumor Segmentation Benchmark (LiTS). arXiv:1901.04056. doi: 10.48550/arXiv.1901.04056 (2019).

[37] Schenk A Prause G Peitgen HO. Efficient semiautomatic segmentation of 3D objects in medical images. In: Delp SL DiGioia AM Jaramaz B, editors. MICCAI, LNCS (1935). Berlin: Springer (2000). p. 186–95.

[38] Felix T Kock F Hänsch A Georgii J Abolmaali N Endo I Improving deep learning based liver vessel segmentation using automated connectivity analysis. In: Colliot O Išgum I, editors. Proceedings of SPIE, vol. 12032. Bellingham: SPIE (2022). p. 120323E. doi: 10.1117/12.2612526.

[39] Kock F Chlebus G Thielke F Schenk A Meine H. Hepatic artery segmentation with 3D convolutional neural networks. In: Drukker K Iftekharuddin KM Lu H Mazurowski MA Muramatsu C Samala RK, editors. Proceedings of SPIE, vol. 12033. Bellingham: SPIE (2022). p. 120331O. doi: 10.1117/12.2607253.

[40] Fasel JHD Schenk A. Concepts for liver segment classification: neither old ones nor new ones, but a comprehensive one. J Clin Imaging Sci. (2013) 3(1):48–54. 10.4103/2156-7514.120803 24228216 PMC3823389

[41] Radtke A Sotiropoulos GC Molmenti EP Schroeder T Peitgen HO Frilling A Computer-assisted surgery planning for complex liver resections: when is it helpful? A single-center experience over an 8-year period. Ann Surg. (2010) 252(5):876–83. 10.1097/SLA.0b013e3181fdd012 21037445

[42] Lang H Radtke A Hindennach M Schroeder T Frühauf NR Malago M Impact of virtual tumor resection and computer-assisted risk analysis on operation planning and intraoperative strategy in major hepatic resection. Arch. Surg. (2005) 140(7):629–38. 10.1001/archsurg.140.7.629 16027326

[43] Yoon JH Choi J-I Jeong YY Schenk A Chen L Laue H Pre-treatment estimation of future remnant liver function using gadoxetic acid MRI in patients with HCC. J Hepatol. (2016) 65(6):1155–62. 10.1016/j.jhep.2016.07.024 27476767

[44] van Ginneken B. Fifty years of computer analysis in chest imaging: rule-based, machine learn-ing, deep learning. Radiol Phys Technol. (2017) 10(1):23–32. 10.1007/s12194-017-0394-5 28211015 PMC5337239

[45] Astley JR Wild JM Tahir BA. Deep learning in structural and functional lung image analysis. Br J Radiol. (2022) 95:1132. 10.1259/bjr.20201107 PMC9153705 33877878

[46] Lassen-Schmidt B Hering A Krass S Meine H. Automatic segmentation of the pulmonary lobes with a 3D u-net and optimized loss function. Med Imaging Deep Learn. (2020). 10.48550/arXiv.2006.00083

